# Taming the High – Are There Stabilizing Medications for Stimulant Use Disorder?

**DOI:** 10.1192/bjo.2026.11784

**Published:** 2026-06-30

**Authors:** New Fei Ho, Song Guo

**Affiliations:** Institute of Mental Health, Singapore, Singapore

## Abstract

**Aims:**

No pharmacological stabilising treatments are currently approved for stimulant use disorder, despite its rapidly increasing global prevalence. Stimulants–including methamphetamine, amphetamine, and cocaine–continue to rise in production and availability worldwide. Methamphetamine remains one of the most misused Class A drugs. In Singapore, Methamphetamine–commonly known as “ICE”–is the leading drug implicated in arrests. In the absence of effective treatments, patients’ lived experiences may provide insights into potential therapeutic strategies.

**Methods:**

We describe two cases illustrating non-prescription use of Armodafinil among individuals with methamphetamine use disorder.

Mr M, a 40-year-old Indian man with methamphetamine use disorder with three prior incarcerations for methamphetamine-related offences, presented to the emergency department with methamphetamine withdrawal symptoms and poor sleep. He reported intermittent use of ICE over the preceding three months. He disclosed substituting with armodafinil when unable to obtain ICE from his usual supplier. He described armodafinil as improving focus and mood, enabling him to function in daily activities, including his work as a cleaner, without producing the euphoric “high” associated with methamphetamine.

Mr N, a 54-year-old Malay man with methamphetamine use disorder, sedative–hypnotic use disorder, and prior drug-induced psychotic episodes, was brought to clinic by his cousin. He presented with acute psychotic symptoms, including auditory and visual hallucinations and grandiose delusions, along with insomnia of two days. After stabilisation with antipsychotic treatment, he denied recent ICE use but disclosed regular armodafinil use over several months. He reported ingesting approximately 500 mg of daily dose, purchased illicitly, to maintain alertness and daily functioning. He described armodafinil as more effective and longer-lasting than caffeinated beverages and denied cravings comparable to ICE.

**Results:**

Chronic stimulant exposure dysregulates dopaminergic and noradrenergic neurotransmission, resulting in blunted reward processing, and compulsive drug-seeking. Modafinil and armodafinil, approved for sleep–wake disorders, act via atypical dopamine transporter inhibition. Modafinil has been explored as a substitution agent in stimulant use disorder, though randomised controlled trials have not demonstrated clear relapse-prevention efficacy. There has been no trials of armodafinil yet. Mechanistically, armodafinil is more potent and longer-acting than modafinil. This case study shows its risks, including psychosis and insomnia, particularly at high or unsupervised doses.

**Conclusion:**

These cases highlight an emergent, patient-driven harm-reduction pattern in which Armodafinil is used as a functional substitute for methamphetamine to preserve wakefulness and daily functioning. This behaviour underscores the unmet need for stabilising pharmacological options in stimulant use disorder and warrants further systematic investigations.

